# Procoagulatory changes induced by head-up tilt test in patients with syncope: observational study

**DOI:** 10.1186/s12959-017-0139-z

**Published:** 2017-06-20

**Authors:** Viktor Hamrefors, Artur Fedorowski, Karin Strandberg, Richard Sutton, Nazim Isma

**Affiliations:** 1 Department of Clinical Sciences Malmö, Lund University, SE 205-02 Malmö, Sweden; 2 Department of Internal Medicine, Skåne University Hospital, SE 205-02 Malmö, Sweden; 3 Department of Medical Imaging and Physiology, Skåne University Hospital, SE 205-02 Malmö, Sweden; 4 Department of Cardiology, Skåne University Hospital, Inga Marie Nilssons gata 46, SE 205-02 Malmö, Sweden; 5 Centre for Thrombosis and Haemostasis, Skåne University Hospital, SE 205-02 Malmö, Sweden; 6 National Heart and Lung Institute, Imperial College, Hammersmith Hospital Campus, Ducane Road, London, W12 0NN UK; 7 Department of Cardiology, Skåne University Hospital, SE 221-85 Lund, Sweden

**Keywords:** Orthostatic stress, Hypercoagulability, Coagulation factors, Partial thromboplastin time, Fibrinogen, von Willebrand factor

## Abstract

**Background:**

Orthostatic hypercoagulability is proposed as a mechanism promoting cardiovascular and thromboembolic events after awakening and during prolonged orthostasis.

We evaluated early changes in coagulation biomarkers induced by tilt testing among patients investigated for suspected syncope, aiming to test the hypothesis that orthostatic challenge evokes procoagulatory changes to a different degree according to diagnosis.

**Methods:**

One-hundred-and-seventy-eight consecutive patients (age, 51 ± 21 years; 46% men) were analysed. Blood samples were collected during supine rest and after 3 min of 70° head-up tilt test (HUT) for determination of fibrinogen, von Willebrand factor antigen (VWF:Ag) and activity (VWF:GP1bA), factor VIII (FVIII:C), lupus anticoagulant (LA1), functional APC-resistance, and activated prothrombin time (APTT) with and without activated protein C (C+/−). Analyses were stratified according to age, sex and diagnosis.

**Results:**

After 3 min in the upright position, VWF:Ag (1.28 ± 0.55 vs. 1.22 ± 0.54; *p* < 0.001) and fibrinogen (2.84 ± 0.60 vs. 2.75 ± 0.60, *p* < 0.001) increased, whereas APTT/C+/− (75.1 ± 18.8 vs. 84.3 ± 19.6 s; *p* < 0.001, and 30.8 ± 3.7 vs. 32.1 ± 3.8 s; *p* < 0.001, respectively) and APC-resistance (2.42 ± 0.43 vs. 2.60 ± 0.41, *p* < 0.001) decreased compared with supine values. Significant changes in fibrinogen were restricted to women (*p* < 0.001) who also had lower LA1 during HUT (*p* = 0.007), indicating increased coagulability. Diagnosis vasovagal syncope was associated with less increase in VWF:Ag during HUT compared to other diagnoses (0.01 ± 0.16 vs. 0.09 ± 0.17; *p* = 0.004).

**Conclusions:**

Procoagulatory changes in haemostatic plasma components are observed early during orthostasis in patients with history of syncope, irrespective of syncope aetiology. These findings may contribute to the understanding of orthostatic hypercoagulability and chronobiology of cardiovascular disease.

## Background

It has long been observed that major cardiovascular and thromboembolic events show higher incidence after awakening, which has been partly explained by increased platelet activity during morning hours [1–4]. In parallel, a phenomenon of “orthostatic hypercoagulability”, i.e. increase in plasma procoagulants independent of postural volume shift and extravascular fluid escape, has been reported [5–7]. The prothrombotic changes during passive orthostasis were first detected in healthy volunteers without history of syncope, cardiovascular or thromboembolic disease. Similar changes in coagulation factors were induced by lower-body negative pressure in volunteers who developed vasovagal reflex but, interestingly, those who did not develop the reflex showed no significant alterations in coagulation factors [8]. Changes in the coagulation system have also been detected during vasovagal reflex activation: a study performed in subjects with von Willebrand disease has demonstrated increased antigen concentration of von Willebrand factor (VWF:Ag), VWF-Ristocetin-cofactor, and factor VIII activity (FVIII:C) after fainting prompted by fear of venepuncture [9]. In a previous study, we have observed that von Willebrand factor is increased in patients with orthostatic hypotension compared with other patients investigated for suspected syncope, irrespective of body position [10]. However, we have also noticed that both groups demonstrated procoagulatory changes during passive orthostasis evoked by head-up tilt test (HUT). In this study, we took the opportunity to analyse plasma samples collected at rest and during the early phase of HUT to assess how passive orthostasis impacts coagulation factors. We aimed to test the hypothesis that passive orthostasis during HUT would evoke procoagulatory changes and that these changes would differ according to HUT diagnosis.

## Methods

### Patients and inclusion/exclusion criteria

The present study was performed from November 2011 to October 2012 as a part of the ongoing Syncope Study of Unselected Population in Malmo (SYSTEMA) project, a single-centre observational study on syncope aetiology [11]. During this period, 233 consecutive patients underwent head-up tilt test (HUT) due to unexplained syncope and/or orthostatic intolerance. Patients were included if they accepted blood sampling during HUT (*n* = 183), and excluded if they were on current oral anti-coagulation therapy with warfarin (*n* = 5), leaving 178 patients that were eligible for the final inclusion. None of the included patients had a diagnosed bleeding disorder. All patients provided written informed consent and were included in further analyses. The Regional Ethical Review Board of Lund University approved the study protocol including blood sampling during tilt testing.

### Head-up tilt test

Study participants were requested to take their regular medication and fast for 2 h before the test, although they were allowed to drink water. The examination was based on a specially designed HUT protocol, which included peripheral vein cannulation, supine rest for 15 min and HUT with optional nitroglycerine provocation after 20 min of passive HUT according to the Italian protocol [12]. Beat-to-beat blood pressure (BP) and ECG were continuously recorded using a Nexfin monitor (BMEYE, Amsterdam, The Netherlands) [13]. The detailed description of the examination protocol has been previously published [14]. The final HUT diagnoses were concordant with the current European Society of Cardiology guidelines [15].

### Blood sampling

Blood was collected during supine rest before and after 3 min of 70° HUT (during passive phase, prior to optional nitroglycerine administration) in citrated tubes (BD Vacutainer®, 4,5 mL, 0,109 M sodium citrate). Samples were immediately centrifuged for 20 min at 2000 x g. Plasma was separated and immediately frozen at −70 °C.

### Coagulation analyses

APTT was performed at the accredited hospital laboratory using a BCS-XP analyzer (Siemens Healthcare, Marburg, Germany) with the Actin FSL reagents (Siemens Healthcare, Marburg, Germany), with and without addition of activated protein C (reference interval in healthy individuals for test without activated protein C, 26–33 s). The analysis of plasma fibrinogen concentration was performed using the Dade Thrombin reagent on a Sysmex CS-5100 analyser (Siemens). The reference interval has been established locally as 2–4 g/L. The coagulation analyses below were performed on a BCS-XP instrument (Siemens). The original plasma-based activated protein C resistance (APC-resistance) test was performed with COATEST APC Resistance test (Chromogenix, Mölndal, Sweden). Reference interval was >2.1. The activity of von Willebrand factor (VWF:GP1bA) was analysed with an immunoassay based on an antibody against glycoprotein 1b (GP1b) and a GP1b construct that binds VWF in the sample (Innovance VWF:Ac; Siemens). Reference interval established locally was 0,5–2.0 kU/L. Antigen concentration of von Willebrand factor (VWF:Ag) was measured with a latex-enhanced immunoassays (Siemens). Reference interval was 0.6–2.7 kIU/L. Screening test for lupus anticoagulant (LA1) was performed with a clot-based assay: LA1 (Siemens). Reference interval was <42 s. Factor VIII activity assay (FVIII:C) was performed with a chromogenic substrate method (Coatest FVIII SP, Chromogenic). Reference interval was 0.5–2.0 kIU/L. Imprecision measured as a coefficient of variation was 2% for VWF:Ag (at 1.2 kIU/L), 3% for VWF:GP1bA (at 0.9 kIU/L), 4% for LA1: (at 40 s), 3% for FVIII: (at 0.8 and 1.5 kIU/L), and 2–4% for fibrinogen (at 1 g/L, 2%; at 3 g/L, 4%).

### Statistical analyses

Independent samples Student’s t-test was used to compare continuous variables between men and women whereas paired samples Student’s t-test was used to compare changes in coagulation parameters measured in supine position and during HUT, respectively. Analyses regarding coagulation parameters were stratified according to sex and age (younger vs. older adults; 65 years). Changes in coagulation parameters during HUT according to diagnosis were specifically tested by comparing the changes in subjects with VVS versus all other diagnoses (including negative HUT) and comparing the changes in subjects with OH (classical + delayed form) versus all other diagnoses, using independent samples Student’s t-test. Categorical variables were compared using Pearson’s chi-square test. All analyses were performed using IBM SPSS Statistics version 23 (SPSS Inc., Chicago, IL, USA). All tests were two-sided whereby *p* < 0.05 was considered statistically significant.

## Results

Patient characteristics stratified by gender are shown in Table 1. Men were older and had lower resting heart rate. Sixty-nine (38.8%) patients were diagnosed with vasovagal reflex syncope (VVS), whereas 49 (27.5%) patients met OH criteria. Further, 7 patients had carotid sinus syndrome, 7 postural orthostatic tachycardia syndrome, 9 initial OH, 5 psychogenic pseudosyncope and 17 (9.6%) underwent HUT without a definitive diagnosis (i.e. negative test). Table 2 shows data on measurements of coagulation markers in supine and after 3 min of HUT. All patients had a significantly increased level of VWF:Ag and fibrinogen after 3 min of HUT, whereas APC-resistance ratio and APTT were significantly decreased compared with resting supine values. There were no significant differences between supine and HUT regarding FVIII, VWF:GP1bA and LA1. All parameters were within the reference interval for healthy individuals. In gender-stratified analyses, only women had significantly increased fibrinogen and demonstrated a shorter LA1 time during HUT (see: Table 3). Stratification of study population by the age of 65 years showed similar distribution of changes in coagulation parameters (Table 4). A final diagnosis of VVS was associated with less increase in VWF:Ag during orthostasis (0.01 ± 0.16) compared to all other diagnoses (0.09 ± 0.17; *p* = 0.004). The changes in coagulation parameters were not different in patients diagnosed with OH compared with all other diagnoses (*p* > 0.05).

**Table 1:** Characteristics and risk factors in 178 consecutive patients with unexplained syncope, mean ± SD or n (%)

	All patients	Men	Women	*p*-value
	*n* = 178	*n* = 81 (46)	*n* = 97 (54)	
*Patient characteristics*				
Age (years)	50.9 ± 20.9	56.0 ± 18.7	46.6 ± 21.8	0.003
BMI (kg/ m2)	25.6 ± 4.6	26.0 ± 3.6	25.3 ± 5.3	0.34
Systolic BP (mmHg)	131 ± 18.7	131 ± 17.1	132 ± 20.0	0.78
Diastolic BP (mmHg)	71 ± 8.0	71 ± 8.8	70 ± 7.6	0.44
Heart rate (bpm)	67.7 ± 11.7	65.4 ± 12.7	69.6 ± 10.6	0.016
*Risk factors (%)*				
Hypertension	28.8	35.0	23.7	0.10
Diabetes mellitus	6.7	8.6	5.2	0.36
History of MACE^a^	3.9	7.4	1.0	0.029
History of stroke	1.7	2.5	1.0	0.46
Present tobacco use	15.7	16.0	15.5	0.92

BMI, body mass index; BP, blood pressure; MACE^a^ (=major coronary event) was defined as history of myocardial infarction, unstable angina, CABG, or PCI

**Table 2:** Coagulation markers in supine and after 3 min head-up tilt (HUT) in 178 patients with unexplained syncope, mean ± SD or n (%)

	All patients	*p*-value*	Men	Women	*p*-value¶
N (%)	178		81 (46)	97 (54)	
FVIII:C *supine* (kIE/L)	1.06 ± 0.38	0.43	1.07 ± 0.32	1.04 ± 0.41	0.56
FVIII:C *HUT* (kIE/L)	1.07 ± 0.36		1.07 ± 0.35	1.07 ± 0.36	0.98
VWF:Ag *supine* (kIE/L)	1.22 ± 0.54	<0.001	1.28 ± 0.56	1.17 ± 0.51	0.17
VWF:Ag *HUT* (kIE/L)	1.28 ± 0.55		1.33 ± 0.59	1.24 ± 0.52	0.31
VWF:GP1bA *supine* (kIE/L)	1.24 ± 0.55	0.20	1.30 ± 0.59	1.19 ± 0.52	0.19
VWF:GP1bA *HUT* (kIE/L)	1.26 ± 0.56		1.31 ± 0.64	1.23 ± 0.49	0.34
Fibrinogen *supine* (g/L)	2.75 ± 0.60	<0.001	2.67 ± 0.57	2.81 ± 0.62	0.11
Fibrinogen *HUT* (g/L)	2.84 ± 0.60		2.73 ± 0.59	2.93 ± 0.60	0.022
LA1 *supine* (sec)	31.2 ± 3.7	0.066	31.9 ± 3.50	30.7 ± 3.8	0.027
LA1 *HUT* (sec)	31.1 ± 4.7		32.0 ± 3.92	30.5 ± 5.2	0.030
APC-resistance *supine*	2.60 ± 0.41	<0.001	2.65 ± 0.39	2.57 ± 0.42	0.17
APC-resistance *HUT*	2.42 ± 0.43		2.49 ± 0.41	2.36 ± 0.44	0.044
APTT/C+ *supine* (sec)	84.3 ± 19.6	<0.001	85.8 ± 19.8	83.1 ± 19.5	0.36
APTT/C+ *HUT* (sec)	75.1 ± 18.8		77.4 ± 18.7	73.1 ± 18.7	0.13
APTT/C- *supine* (sec)	32.1 ± 3.8	<0.001	32.1 ± 4.1	32.1 ± 3.6	1.0
APTT/C- *HUT* (sec)	30.8 ± 3.7		30.8 ± 3.8	30.7 ± 3.7	0.83

*supine vs. HUT paired samples t-test; ¶ men vs. women independent samples t-test; FVIII, factor VIII; VWF:Ag, von Willebrand factor antigen; vVWF:GP1bA, von Willebrand factor GP1bA activity; LA1, lupus anticoagulant-screening; APC-resistance, activated protein C resistance; APTT, activated prothrombin time; C+/−, with/without activated protein C

**Table 3:** Gender-stratified changes in coagulation markers during head-up tilt among 178 patients with unexplained syncope

	Men	*p*-value*	Women	*p*-value*
N (%)	81 (46)		97 (54)	
Δ FVIII:C (kIE/L)	0.00 ± 0.16	0.64	0.02 ± 0.13	0.091
Δ VWF:Ag (kIE/L)	0.04 ± 0.18	0.030	0.07 ± 0.15	<0.001
Δ VWF:GP1bA (kIE/L)	0.01 ± 0.25	0.86	0.03 ± 0.18	0.067
Δ Fibrinogen (g/L)	0.06 ± 0.32	0.12	0.12 ± 0.22	<0.001
Δ LA1 (sec)	−0.13 ± 3.23	0.72	−0.56 ± 1.96	0.007
Δ APC-resistance	−0.16 ± 0.26	<0.001	−0.21 ± 0.26	<0.001
Δ APTT/C+ (sec)	−8.3 ± 13.7	<0.001	−9.9 ± 10.6	<0.001
Δ APTT/C- (sec)	−1.2 ± 2.4	<0.001	−1.4 ± 1.7	<0.001

*supine vs. HUT paired samples t-test; FVIII, factor VIII; VWF:Ag, von Willebrand factor antigen; vWF:GP1bA, von Willebrand factor GP1bA activity; LA1, lupus anticoagulant-screening; APC-resistance, activated protein C resistance; APTT, activated prothrombin time; C+/−, with/without activated protein C

**Table 4:** Age-stratified changes in coagulation markers during head-up tilt among 178 patients with unexplained syncope

	Age < 65 years	*p*-value*	Age ≥ 65 years	*p*-value*
N (%)	119 (67)		59 (33)	
Δ FVIII:C (kIE/L)	0.02 ± 0.15	0.17	−0.01 ± 0.15	0.55
Δ VWF:Ag (kIE/L)	0.04 ± 0.15	0.006	0.10 ± 0.19	<0.001
Δ VWF:GP1bA (kIE/L)	0.02 ± 0.22	0.35	0.02 ± 0.19	0.37
Δ Fibrinogen (g/L)	0.10 ± 0.30	<0.001	0.07 ± 0.20	0.009
Δ LA1 (sec)	−0.40 ± 2.88	0.14	−0.31 ± 1.92	0.25
Δ APC-resistance	−0.16 ± 0.24	<0.001	−0.24 ± 0.28	<0.001
Δ APTT/C+ (sec)	−8.5 ± 12.2	<0.001	−10.6 ± 11.9	<0.001
Δ APTT/C- (sec)	−1.3 ± 2.1	<0.001	−1.3 ± 2.0	<0.001

*supine vs. HUT paired samples t-test; FVIII, factor VIII; VWF:Ag, von Willebrand factor antigen; vWF:GP1bA, von Willebrand factor GP1bA activity; LA1, lupus anticoagulant-screening; APC-resistance, activated protein C resistance; APTT, activated prothrombin time; C+/−, with/without activated protein C

## Discussion

The present study illustrates the impact of passive orthostatic challenge on haemostatic plasma components in a series of unselected patients with a history of unexplained syncope. During early phase of head-up tilt test, fibrinogen, APTT, VWF:Ag, and APC-resistance demonstrated significant procoagulatory changes. Although relative changes in the assessed coagulation markers were small, indeed, within the test imprecision range for fibrinogen, the directionality of these changes consequently pointed at the increased coagulability on standing.

Fibrinogen, as acute-phase glycoprotein, may be elevated in any form of inflammation and is also involved in formation of blood clots not only through fibrin-formation, but also through its effect on specific platelet receptors and blood viscosity. Higher levels of fibrinogen are associated with cardiovascular disease [16–19] and its degradation products accumulate in the atherosclerotic plaque [20]. Interestingly, fibrinogen increased most in women who also demonstrated procoagulatory LA1 changes. The exact mechanisms behind these gender-specific propensities are not known and were not the subject of this study, however, hormonal differences may be one of the possible explanations [21]. Thus, orthostatic procoagulatory changes in fibrinogen, LA1, and FVIII seem mainly to affect women who are at higher risk of thromboembolic events compared with men [22]. As thromboembolic events are more common in the morning hours [3], orthostatic increase in fibrinogen and FVIII may act as a facilitator of thrombus generation after awakening and assuming an upright position, in a relative dehydrated state after a night’s rest.

Notably, APTT was reduced on orthostasis irrespective of the analysed subgroup. Shortening of APTT usually indicates increased activity of FVIII in association with inflammatory conditions [23]. As APTT involves both fibrinogen and FVIII, which first and foremost increased in women, it seems that other factors such as prothrombin, V, IX, X, XI, and XII may have undergone orthostatic changes too. Unfortunately, our test panel did not detect this. Shortening of APTT, although within the normal range, may have thromboembolic consequences, as indicated by a previous study [24]. We believe that shortened APTT indicates a hypercoagulable state, as was also shown in a study by Mina et al. [25]. This is supported by other clinical studies performed on patients with acute coronary events [26, 27].

Furthermore, we observed elevated levels of VWF during orthostasis. It has been reported that higher levels of VWF are associated with a 3-fold increased risk for severe coronary heart disease [28–30]. Our results are consistent with other studies, linking changes in VWF and FVIII to the vasovagal reflex, artificially induced orthostatic stress and presyncope [5, 6, 8]. The von Willebrand factor is the largest plasma protein and plays a key role in primary haemostasis as cofactor in platelet adhesion and platelet aggregation. This platelet adhesion is promoted by a platelet membrane receptor glycoprotein (GPIb –IX-V) when circulating VWF attaches to the sub-endothelium collagen and serves as a bridge between tissue and platelets [31].

Previous studies performed on healthy subjects have shown that platelet aggregability is increased in the morning, when the incidence of cardiovascular events such as myocardial infarct, sudden cardiac death and transient myocardial ischemia is higher [1, 32–35]. However, data on the changes in haemostatic plasma components in the same settings are very sparse and we believe that this study may contribute to the understanding of cardiovascular chronobiology. Our results confirm that changing from supine to upright body position induces activation of the coagulation system and promotes a hypercoagulable state during early orthostasis. The physiological role of orthostatic hypercoagulability may be to protect the body against increased bleeding risk during activities of daily life or be a result of higher hydrostatic pressure in the lower body, which will activate the endothelial response and release of coagulation factors.

The activation of haemostatic plasma components may support the hypothesis of tight cooperation between procoagulatory factors and platelets, and its impact on the development of cardiovascular events. Whether or not cardiovascular events such as myocardial infarct, sudden cardiac death, transient myocardial ischemia, and stroke during morning hours are promoted by synergic effects of both orthostatic hypercoagulability and increased platelet aggregability remains to be further explored.

Finally, patients diagnosed with vasovagal reflex syncope (VVS) demonstrated less pronounced changes in vWF:Ag than the rest of study population, whereas those with OH did not differ from non-OH patients. The former may be due to relatively younger age of VVS patients, although this study does not allow further speculations on the cause of this difference, whereas the latter implies that procoagulatory orthostatic changes are independent of BP fluctuations on standing.

### Limitations

The conclusions of our study may be underpowered due to some important limitations (listed below). Thus, we would like to emphasize that the current study may be useful as a “proof of concept” for a larger and improved study design, in which these limitations could be overcome. No control group including healthy individuals was included and there were no measurements of coagulation parameters in subjects that did not undergo HUT. As the participants of the current study are part of the ongoing larger SYSTEMA project [11], no prospective sample size assessment was done, prior to the study. We were not able to measure specific markers of activated coagulation, such as thrombin-antithrombin-complex (TAT) or D-dimer, which would have been informative. Furthermore, since data on hereditary thrombophilia and levels or function of platelets were not registered, it was not feasible to separate possible contributions of these variables to hypercoagulability. Procoagulants were measured after 3 min of HUT only and we do not have data on how the prolonged standing might influence the assessed coagulation factors. However, determination of early orthostatic changes may have precluded the possible effects of intravascular volume escape observed during prolonged standing on the concentration of procoagulatory proteins. Also of relevance, even though the observed rise in coagulation markers is in direction during HUT, we cannot exclude a similar reaction if subjects were to be provoked by physical stress that did not involve orthostasis (such as on a supine ergometer bicycle). Finally, our study design and the fact that we did not measure potentially important coagulation proteases such as thrombin, factor Xa and tissue factor involved in atherosclerosis development, precludes us from drawing any conclusions on whether syncope induced hypercoagulability might also contribute to the development of atherosclerosis.

## Conclusions

Procoagulatory changes in haemostatic plasma components can be observed during early phase of passive orthostatic challenge irrespective of syncope diagnosis. These findings may contribute to the understanding of orthostatic hypercoagulability and chronobiology of cardiovascular disease.

## Abbreviations

APC-resistance: Activated protein C resistance
APTT: Activated partial thromboplastin time
FVIII:C: Factor VIII activity
HUT: Head-up tilt test
LA1: Lupus anticoagulant
OH: Orthostatic hypotension
VVS: Vasovagal syncope
VWF:Ag: Antigen concentration of von Willebrand factor
VWF:GP1bA: Activity of von Willebrand factor
